# Physical Activity during Cancer Treatment (PACT) Study: design of a randomised clinical trial

**DOI:** 10.1186/1471-2407-10-272

**Published:** 2010-06-09

**Authors:** Miranda J Velthuis, Anne M May, Ria AG Koppejan-Rensenbrink, Brigitte CM Gijsen, Eric van Breda, G Ardine de Wit, Carin D Schröder, Evelyn M Monninkhof, Eline Lindeman, Elsken van der Wall, Petra HM Peeters

**Affiliations:** 1 Comprehensive Cancer Center Middle Netherlands, Utrecht, the Netherlands; 2 Julius Center for Health Sciences and Primary Care, University Medical Center, Utrecht, the Netherlands; 3 Comprehensive Cancer Center Limburg, Maastricht, the Netherlands; 4 School for Nutrition, Toxicology and Metabolism, Maastricht University Medical Centre, Maastricht, the Netherlands; 5 Department of Movement Sciences Maastricht University, Maastricht, the Netherlands; 6 National Institute of Public Health and the Environment, Bilthoven, the Netherlands; 7 Rehabilitation Center De Hoogstraat, Utrecht, the Netherlands; 8 Educational Center for Musculoskeletal Therapies, SOMT, Amersfoort, the Netherlands; 9 Rudolf Magnus Institute of Neurosciences, University Medical Center Utrecht, Utrecht, the Netherlands; 10 University Medical Center Utrecht, Utrecht, the Netherlands

## Abstract

**Background:**

Fatigue is a major problem of cancer patients. Thirty percent of cancer survivors report serious fatigue three years after finishing treatment. There is evidence that physical exercise during cancer treatment reduces fatigue. This may also lead to an improvement of quality of life. Such findings may result in a decrease of healthcare related expenditures and societal costs due to sick leave. However, no studies are known that investigated these hypotheses. Therefore, the primary aim of our study is to assess the effect of exercise during cancer treatment on reducing complaints of fatigue and on reducing health service utilisation and sick leave.

**Methods/Design:**

The Physical Activity during Cancer Treatment study is a multicentre randomised controlled trial in 150 breast and 150 colon cancer patients undergoing cancer treatment. Participants will be randomised to an exercise or a control group. In addition to the usual care, the exercise group will participate in an 18-week supervised group exercise programme. The control group will be asked to maintain their habitual physical activity pattern. Study endpoints will be assessed after 18 weeks (short term) and after 9 months (long term). Validated questionnaires will be used. Primary outcome: fatigue (Multidimensional Fatigue Inventory and Fatigue Quality List) and cost-effectiveness, health service utilisation and sick leave. Secondary outcome: health related quality of life (European Organisation Research and Treatment of Cancer-Quality of Life questionnaire-C30, Short Form 36 healthy survey), impact on functioning and autonomy (Impact on functioning and autonomy questionnaire), anxiety and depression (Hospital Anxiety and Depression Scale), physical fitness (aerobic peak capacity, muscle strength), body composition and cognitive-behavioural aspects. To register health service utilisation and sick leave, participants will keep diaries including the EuroQuol-5D. Physical activity level will be measured using the Short Questionnaire to Assess Health-Enhancing Physical Activity and will be monitored with an exercise log and a pedometer.

**Discussion:**

This study investigates the (cost)-effectiveness of exercise during adjuvant treatment of patients with breast or colon cancer. If early physical exercise proves to be (cost) effective, establishing standardised physical exercise programmes during cancer treatment will be planned.

**Trial registration:**

Current Controlled trials ISRCTN43801571, Dutch Trial Register NTR2138

## Background

With the rising number of cancer survivors, there is an increasing awareness of maintaining optimal health in this group. Although treatment of other cancer- or treatment-related symptoms such as nausea and pain has improved considerably during the last decades, there is still no accepted treatment for complaints of fatigue. Sixty to 96% of (former) cancer patients report complaints of fatigue during and/or after treatment [1,2]. Although levels of fatigue decrease gradually in disease-free survivors, 30% of cancer survivors still report serious complaints of fatigue three years after completion of medical treatment [3]. Fatigue is expected to lead to decreased quality of life, decreased levels of physical activity and increased episodes of sick leave and production loss. For instance, in 2005, in the Netherlands 22.000 persons were unfit for work due to current or previous cancer [4].

The cause of fatigue in cancer patients and survivors is unknown: it may be caused by the disease itself, its treatment or it may be the result of psychological and physiological responses [2]. Deconditioning due to further reduction of physical activity in cancer patients might even further affect feelings of fatigue [5]. The National Comprehensive Cancer Network, an alliance of 21 of the world's leading cancer centres, advises to start physical exercise shortly after cancer diagnosis [6]. Several studies, mainly small scaled and in breast cancer patients, have shown that exercise during cancer treatment prevents complaints of fatigue and improves quality of life [7-13]. A Cochrane meta-analysis recently provided evidence that exercise is beneficial in the management of cancer-related fatigue, also during cancer treatment [12]. The meta-analysis includes physical exercise during or after cancer treatment, in adults, regardless of gender, age, tumour type, tumour stage or type of cancer treatment. Interventions took place in different settings and included all types of exercise (supervised as well as home-based exercise and aerobic as well as resistance exercise). We published a meta-analysis assessing effects of different exercise prescription parameters during adjuvant treatment on cancer related fatigue with special emphasis on safety and feasibility of exercise during adjuvant treatment [13]. Significant beneficial effects were visible for exercise during breast cancer treatment (supervised aerobic), adherence was moderate to excellent and few adverse events occurred.

However, cost-effectiveness of such programmes has not been studied before. We hypothesise that an early start of physical exercise during treatment will lead to a decrease in health care related expenditures by reducing healthcare utilisation. Especially reductions in the number of visits to medical specialists, general practitioners and from home care workers are expected.

We further anticipate that the beneficial effect on healthcare related expenditures is present during the exercise programme, and that it will continue to exist after termination of the physical activity programme. Furthermore, we hypothesise that early physical exercise programmes will lead to a reduction in sick leave and related production loss and that such programmes will be cost-effective. To study this hypothesis, we designed a randomised clinical trial: the Physical Activity during Cancer Treatment (PACT) study. In this manuscript, we describe the design and methods of the PACT study.

## Methods/Design

### Design

The PACT-study examines the effects of exercise during cancer treatment on reducing complaints of fatigue and in reducing health service utilisation and sick leave (primary outcomes). In addition, we will study the effects on improving health related quality of life, impact on autonomy and participation, anxiety and depression, physical fitness, body composition and cognitive-behavioral aspects (secondary outcomes). This study is designed as a multicentre pragmatic randomised controlled trial, with two study arms, i.e. a group invited for an exercise programme in addition to usual care; and the control group receiving usual care while maintaining their habitual physical activity pattern. The exercise programme is an 18-week supervised group programme. Because blinding of participants towards allocation is not feasible, this study has a pragmatic design.

### Subjects

A total of 300 newly diagnosed breast and colon cancer patients (150 per cancer type), who will receive adjuvant treatment, including chemotherapy, will be invited to participate in the PACT-study.

**Figure 1:**
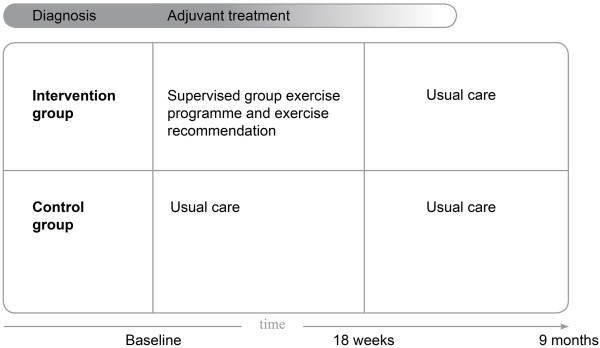
**Interventions and timeline**.

We decided to include patients diagnosed with breast and colon cancer, since these cancer types are among the most prevalent [13,14]. Inclusion criteria are: histological diagnosis of cancer less than six (breast cancer) or ten (colon cancer) weeks before study recruitment; stage M0; and scheduled for chemotherapy. Breast cancer patients with immediate use of a tissue expander after surgery may be included until ten weeks after histological diagnosis. They will start the exercise programme after replacement of the tissue expander by a breast prosthesis, since exercising with a tissue expander is discouraged by medical specialists. In agreement with the recommendations by the National Comprehensive Cancer Network, patients allocated to the exercise group will start shortly after diagnosis [6]. We decided to recruit patients scheduled for chemotherapy, because medical specialists frequently report fatigue in these patients. Some patients are also scheduled for radiotherapy preceding the chemotherapy. By choosing a more or less arbitrarily 18-week exercise programme, the programme in these patients will at least run during part of their chemotherapy treatment (see Figure 1).

Additional inclusion criteria are: age 25-75 years; not treated for cancer in the five years preceding recruitment (except basal skin cancer); able to read and understand the Dutch language; Karnovsky Performance Status of 60 or higher; able to walk 100 meter or more; no contra-indications for physical activity (assessed through the Revised Physical Activity Readiness Questionnaire).

Written informed consent will be obtained from all patients. This study has been approved by the Medical Ethics Committee of the University Medical Centre Utrecht and the local Ethical Boards of the participating hospitals.

### Recruitment and Allocation

**Figure 2:**
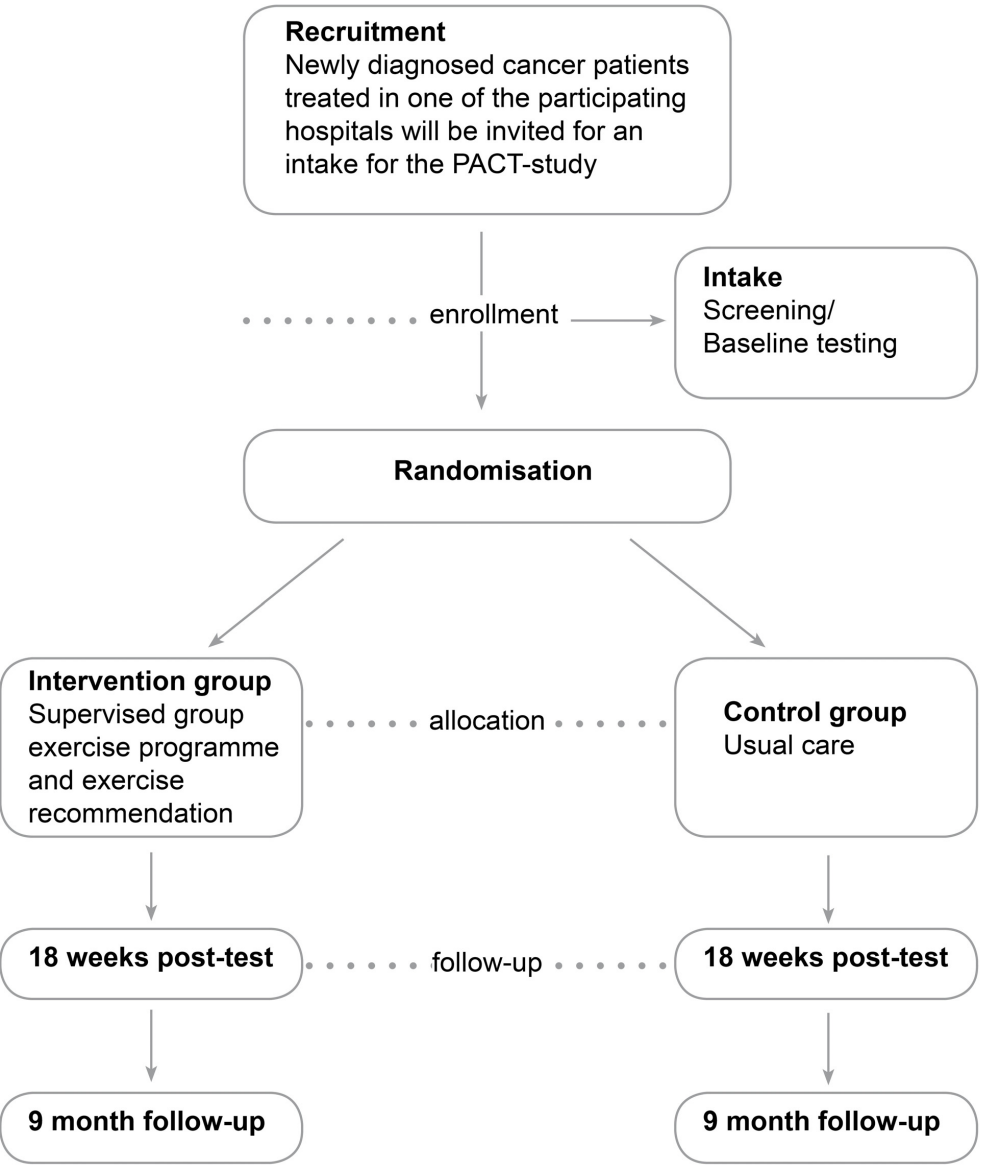
**Planned inclusion and allocation of patients**.

Patients will be recruited by medical specialists and specialised nurses in at least six hospitals in the regions of the Comprehensive Cancer Centers Middle Netherlands, Limburg and Rotterdam. New cancer patients will be informed and invited by the clinician or the oncological nurse during a regular outpatient clinic visit. Patients willing to participate will be asked to visit the study centre to assess study eligibility and for baseline measurements. If eligible, patients will be asked to sign informed consent upon which they will be randomly allocated to the intervention or the control group by central data management. Allocation to the intervention- or control group will be concealed. Randomisation will be stratified per tumour site (breast or colon) by the sequential balancing method. The following characteristics will be balanced: age (25-40, 40-65 and 65-75 years), adjuvant treatment (radiotherapy versus no radiotherapy (before chemotherapy)); using a tissue expander (for breast cancer patients yes versus no) and hospital. For colon cancer patients, gender will be included as a first step in above balancing algorithm. Recruitment and allocation strategy is summarised in Figure 2.

### Exercise Intervention

The exercise programme is designed by applying the principles of 'Intervention Mapping' [15]. Intervention Mapping provides a framework for effective decision making in the developmental process of (health promotion) interventions using five steps: 1) specification of objectives, 2) development of methods and strategies, 3) design of the programme, 4) anticipation of implementation and 5) evaluation. The exercise objectives, specified for the specific target population were set by an in-depth literature search and by seeking advices of medical oncologists, radiotherapists and a surgeon. Subsequently, the exercise methods were developed in close cooperation with physiotherapists experienced in rehabilitation programmes for cancer survivors. This resulted in a clear exercise programme and materials for instruction for physiotherapists as well as information leaflets for patients. We continuously involved medical specialists and physiotherapists in the development in order to optimise implementation of the exercise programme in the health care system.

The exercise programme will be offered at several outpatient clinics of general hospitals. Participants will attend this programme twice a week. The programme incorporates principles for aerobic training and muscle strength training as well as principles of Bandura's social cognitive theory [16].

The programme will be individualised to the patient's personal preferences and physical fitness level. During an intake prior to the exercise programme, information about patient's preferences, regular sports activities, requirements at home and work, and physical fitness level (by means of a symptom limited exercise test and 1-repetition maximum (1-RM) muscle strength tests) and physical limitations will be collected by the physiotherapist. After the intake, the patient will start the 18-week exercise programme. The 1-hour exercise classes will include a warming up (5 min), aerobic and muscle strength training (50 min) and a cooling down (5 min). *Aerobic training *includes interval training of alternating intensity at or below the ventilatory threshold (3 × 2 min increasing to 2 × 7 min) as determined during the baseline symptom limited bicycle ergometry test (see study end points, secondary outcomes). Heart rate and the Borg scale of perceived exertion will be monitored during the aerobic training.

*Muscle strength training *will be performed for all major muscle groups: arms, legs, shoulders, and trunk: 2 × 10 repetitions (65% 1-RM) increasing to 1 × 10 repetitions (75% 1-RM) and 1 × 20 repetitions (45% 1-RM).

The exercise programme incorporates principles of Bandura's social cognitive theory (SCT) to help participants maintaining a physically active lifestyle during and after cancer treatment [16]. This theory emphasises the role of cognitive processes in determining behaviour such as exercise. The most important construct in the theory is self-efficacy that is defined as "an individual's beliefs in his/her capabilities to organise and execute the course of action required to produce given attainments" [16]. The concept has been used successfully in other intervention studies that aim to alter health related behaviour [17]. Self efficacy will be altered using three different techniques, namely actual or mastery experience, vicarious or observational experience and verbal persuasion. First, actual or mastery experience will be used by asking participants to report training results in graphs. Physiotherapists will check these weekly and give positive feedback about the obtained results and will stimulate the participant to make action plans to further increase their exercise level. The starting point of the training schedule has been carefully chosen so it will be likely that the participant will succeed and subsequently have a mastery experience in doing physical exercises.

Vicarious or observational experience is applied by using DVDs where other cancer patients show their experiences with exercise programmes. Also the use of a so-called buddy will help in role modelling as a component of the programme. The buddy is a senior participant introducing a new participant to the programme. During the programme they will work together and support each other when facing difficulties in executing the exercises. Finally, verbal persuasion is used. Physiotherapists will strongly recommend exercise which will be supported by written information.

In addition to the supervised exercise programme twice a week, patients are recommended to be physically active for at least 30 minutes a day, five days a week according to the Dutch guideline for physical activity [18], which is based on international recommendations [19,20]. This should include an aerobic component of moderate intensity in agreement with the participant's fitness and desires.

### Control group

Participants assigned to the control group will receive usual care, i.e., no exercise intervention. They will be asked to maintain their habitual physical activity pattern.

After completion of the present study, the control subjects will be offered participation in a rehabilitation programme for cancer survivors [21-23].

### Study endpoints

At baseline, at 18 weeks and at 9 months study participants will visit the study centre for outcome assessment (see Figure 2). During the entire 9 months, once a week, participants will fill out diaries to assess their healthcare use and work status. Participants will be asked to wear a pedometer during seven days once following baseline measurements and again after 18 weeks and after 9 months. Participants of the exercise group fill out exercise logs. The following data will be collected:

demographics (gender, age, hospital, name of medical specialist) and factors related to cancer (type of cancer; treatment regime). More detailed information regarding diagnosis and the treatment regime will be collected from medical records.

#### Primary outcomes

Fatigue will be measured using the Multidimensional Fatigue Inventory (MFI) and the Fatigue Quality List (FQL) [24,25]. The MFI is a 20-item self-report instrument designed to measure multiple fatigue characteristics and the impact on function. The Dutch version of the MFI has proven to be valid and consists of five subscales (general fatigue, physical fatigue, reduced activity, reduced motivation and mental fatigue) [24].

The FQL consists of 28 adjectives addressing the perception of fatigue [25]. Participants are asked to mark which out of 28 adjectives fit their experienced fatigue. Multiple answers are possible. The adjectives are clustered in four subscales: frustrating, exhausting, pleasant and frightening.

To register health service utilisation and sick leave participants will keep diaries. Diaries will include all types of health care consumption also including contacts with alternative medicine and own out of pocket expenses. Participants will be asked to keep track of their absence from work (if applicable).

The diaries include also a multidimensional measurement of health status, the EuroQuol-5D (EQ-5D). The EQ-5D will be used to calculate quality adjusted life years (QALYs) and consists of the EQ-5D descriptive system and the EQ VAS [26]. The EQ-5D descriptive system comprises five dimensions of health (mobility, self-care, usual activities, pain/discomfort anxiety/depression). Each dimension comprises three levels (no problems, some/moderate problems, extreme problems). The EQ VAS records the respondents self-rated health status on a vertical graduated (0-100) visual analogue scale.

#### Secondary outcomes

To measure health related quality of life the European Organisation Research and Treatment of Cancer-Quality of Life-C30 questionnaire (EORTC-QoL-C30) (version 3) and the Short Form 36 healthy survey (SF-36) will be used. Both questionnaires have been validated [27-29].

The EORTC QOL C30 incorporates five functional scales (physical, role, emotional, cognitive and social functioning), one quality of life scale and one symptom scale (including fatigue and pain). The SF-36 consists of 36 items, organised into eight scales: physical functioning, role limitations due to physical health problems, bodily pain, general health perceptions, vitality, social functioning, role limitations due to emotional problems and general mental health.

The perceived impact of the disease on participation and autonomy will be measured with the validated Impact on Participation and Autonomy (IPA) questionnaire [30,31]. The IPA incorporates 32 items clustered in the subscales autonomy indoors, family role, autonomy outdoors, social life and relationships, and work and education.

Anxiety and depression will be self-rated with the Dutch language version of the Hospital Anxiety and Depression Scale (HADS) [32]. The HADS consists of 14 items, seven items of the depression subscale (HADS-D) and seven items of the anxiety subscale (HADS-A).

Physical fitness will be assessed as aerobic peak capacity and muscle strength. Aerobic capacity will be determined using a symptom-limited bicycle ergometry test with breathing gas analysis using a ramp 10-, 15-, or 20-protocol, dependent on the patient's condition. The load will be increased every minute in such a way that patients reach peak workload within 10 minutes. The test will be terminated on the patients' symptoms or at the physicians' discretion. Borg scores for dyspnoea and muscle fatigue will be taken before and after the test. Peak workload, peak oxygen uptake and Borg scores at peak workload will be taken for analysis. Heart rate and work load at ventilatory threshold will be used to determine the work load for the aerobic training during the exercise programme.

Muscle strength of quadriceps and hamstring will be assessed by using a Cybex dynamometer at 60°/s and 180°/s. After a standardised 5-minute warm-up, five repetitions will be performed to practice before the definitive measurements at 60°/s and 180°/s will be taken. Between all sessions, there will be a 1-minute rest period. The patient will be verbally encouraged. The highest peak torque value of three repetitions for both velocities will be calculated. The fatigue index comparing the first and the last of fifteen repetitions will be calculated at 180°/s.

Handgrip strength of both hands will be measured with a mechanical handgrip dynamometer. The best score of two attempts will be recorded in kilogram force (kgF).

The Body Mass Index (BMI) will be calculated as weight in kilograms divided by height in meters squared (kg/m^2^). Body weight and height (to the nearest 0.5 kg and 0.5 cm respectively) will be measured while the subjects wear light clothes and no shoes using an analogue balance (SECA) and wall mounted tape measure, respectively.

Body fat distribution will be estimated by the waist- and hip circumference. Waist circumference (to the nearest 0.5 cm) will be measured standing at the smallest circumference between abdomen and chest. Hip circumference (to the nearest 0.5 cm) will be measured standing as the largest circumference between waist and thigh. All measurements will be taken in duplicate and averaged.

Self efficacy about the performance of physical activity will be assessed by seven (exercise programme) or eight (exercise recommendation) items based upon the Social Cognitive Theory. Items will be scored on a 5-point Likert scale with endpoints labelled 'strongly disagree' and 'strongly agree'.

Physical activity level will be measured using the Short Questionnaire to assess health enhancing physical activity (SQUASH) [33].

To monitor the physical activity level, participants of the intervention group will also be asked to keep an exercise log during the 18-week exercise programme. In the log, they register the frequency, intensity, and duration of the exercises they were performing during the study period. In addition, physical activity will be measured by a pedometer. Participants, of both the intervention and the control group, will wear the pedometer during seven days following their baseline and follow-up measurements.

The attendance rate for the exercise sessions will be recorded in a Case Record Form.

Adherence to the exercise recommendation will be registered in the exercise log.

Adverse events reported spontaneously by the subject or observed by physiotherapists, study nurse or medical doctor will be recorded, i.e. sports accidents, surmenage, or injuries. All serious adverse events will be reported to the accredited ethical committee that approved the protocol, according to the requirements of that ethical committee.

### Sample size

In accordance with previous studies [34], we consider an effect of a 2-unit improvement in the MFI questionnaire to be clinically relevant. To detect an intervention effect of 2 units of change (± SD 4) in fatigue (MFI questionnaire (range subscale 4-20)) we will need 75 participants in the intervention and control group for each cancer type, 300 participants in total (alpha = 0.05, power = 0.80). A drop-out of 10% is anticipated. The proposed number of participants also allows us to perform analyses for the two types of cancer separately.

This sample is also large enough to detect a clinically relevant intervention effect of 10 units of change (± SD 20) on the secondary outcome EORTC QLQ C30 subscales [35].

### Data analysis

Descriptive statistics will be used to characterise the study population and the instrument scores at baseline and at the follow up measurements. Mean differences including 95% confidence intervals will be calculated. Mean differences will be presented for the total study population and for different subgroups (cancer type, cancer regime). The effect of physical exercise on fatigue will be tested at 18 weeks (end of the exercise programme) and nine months post-enrolment according to the intention to treat principle (primary analyses), and on a per protocol basis including participants of the exercise group who attended at least 75% of the exercise sessions. Longitudinal analyses will be conducted, using mixed linear regression models while taking different levels (time and hospital) into account [35]. In the longitudinal analyses the programme accounts for missing data based on the observed data.

#### Economic Evaluation

In the economic evaluation, the balance between costs and effects will be assessed of a supervised exercise programme versus usual care in cancer patients during treatment. The actual costs incurred with both strategies will be compared up until nine months after randomisation. Results of both cost and effect measurement will be integrated using cost-effectiveness and cost-utility analyses.

Cost estimates will be based on the actual costs, direct and indirect, in both study arms. Direct costs include costs of the exercise programme (estimated to cost approximately €1000 per patient). Other direct costs include costs of health service utilisation such as general practitioner and oncologist contacts, hospitalisation and rehabilitation. We will also register use of alternative medicine, as it is anticipated that part of the patients will use this type of care. Indirect cost include own expenses and travel cost. Furthermore, work status and absence from work due to illness and its treatment will be administrated. Patient will be asked to complete diaries on these direct and indirect cost items. Data from diaries will be summarised at the two follow-up moments. Indirect costs for paid work will be calculated using the friction costs method [31,32].

#### Patient outcome analysis

Intervention and control groups will be compared with regard to the effects of treatment on their health status, using EQ-5D questionnaires. This will enable the calculation of quality adjusted life years gained as a result of the intervention. As the percentage of patients that is expected be fatigued will be reduced from 60-96% to 40-50%, substantial gains in quality of life may be found [6]. Incremental cost-effectiveness ratios (ICER) will be generated by calculating the incremental costs of the exercise programme compared to the non-intervention group divided by the incremental effects [33]. The time horizon of the study will be similar to the study period of nine months. Hence, it will not be necessary to discount costs and life-years.

### Preliminary results

Currently, we conduct a pilot study in three general hospitals and one rehabilitation centre in the region Middle Netherlands to test all major procedures. In order to prevent non-participation in the control group we tested the Zelen randomisation procedure [36]. We hypothesised that cancer patients willing to participate in an exercise trial would be dissatisfied when allocated to usual care only. According to the Zelen design, patients were asked consent only for following them and taking some measurements at baseline, after 18 weeks and nine months. Then after randomisation, only those allocated to the intervention are asked to participate also in the supervised group exercise programme.

One hundred sixty six patients were invited to the study: 12 were not eligible and 90 refused. Reasons amongst others were: long travel distance to the study centre, medical complications, or lack of time. Forty six patients were included, 32 in each study arm. One patient in the control group refused participation (3%) and eight patients in the intervention group (25%). Reasons were again: medical complications (3), long travel distance (2), lack of time (2), other reasons (1). One patient quitted the exercise programme after two sessions (3%). The remaining 23 patients (72%) started the exercise programme and 21 patients (66%) completed the programme. These patients were satisfied with the exercise programme (mean score 8,8 ± SD 0,8 on a 10 point scale) and attended 50 to 97% of all sessions during the 18 week period. Also our testing procedure consisting of several physical fitness tests and questionnaires appeared to be feasible.

We concluded from the pilot study that the Zelen design may have prevented non-participation in the control group, but it seemed to have increased the non-participation in the intervention group. It is likely that women after hearing from the exercise programme were not willing to respond, because they were not prepared for such investment during or after a life event. Because of this, we decided the Zelen design will not be used in the PACT study.

## Discussion

There are several reviews that show that exercise during treatment improves complaints of fatigue [7-13]. However, cost effectiveness of exercise during cancer treatment has not been studied before. Most studies were performed in breast cancer patients. For that reason, we chose to include colon cancer patients as well. Power was calculated to study effects for each cancer site separately.

We will study the effectiveness of a supervised exercise programme that incorporates both aerobic training and muscle strength training as well as cognitive behavioural aspects as suggested by Cramp and Daniel [12]. By incorporating the behavioural aspects of Bandura's social cognitive theory, we expect to increase self efficacy of participants regarding maintaining a physically active lifestyle during and after cancer treatment. Based on the pilot study, we conclude that the exercise programme is feasible for most patients.

However, being also subject of the pilot study, the Zelen randomisation appeared not to be effective. Study subjects willing to participate in the study withdraw because of the unexpected confrontation with the exercise programme. In addition, the logistics of the study were such that many meetings between the women being part of the control group and women participating in the intervention group occurred, e.g. at the hospital during chemo therapy. So, control women soon knew about the exercise programme. Due to these findings, we decided to use conventional randomisation in the definitive PACT study. We expect that the use of the conventional design will lead to a higher inclusion rate. Furthermore, we expanded the PACT-study to more hospitals for patient recruitment. By doing so, we expect to include the aimed 300 patients within two years.

In conclusion, the aim of the PACT-study is to asses the (cost)-effectiveness of exercise during adjuvant treatment of patients with breast or colon cancer. If early physical exercise proves to be effective and cost effective, establishing standardised physical exercise programmes during treatment of breast and colon cancer patients will be a logical next step.

## Competing interests

The authors declare that they have no competing interests.

## Authors' contributions

MV, AKR, BG, EB, EW and PP developed the study concept and initiated the project. An expert group consisting of medical specialists assisted with the specification of the study concept, aims and population. MV drafted the manuscript, participated in the design and coordination of the study. AM and PP advised on drafting the manuscript, participated in the design and coordination of the study. AW advised on the economic evaluation. A working group consisting of physiotherapists experienced in rehabilitation of cancer survivors, assisted in the development of the exercise programme. EB advised on the training schedule for aerobic and muscle strength training of the exercise programme. CS advised on the implementation of cognitive behavioural aspects in the PACT-study. EM and EL advised about the design of the study. All authors revised and approved the final version of the manuscript.

## Pre-publication history

The pre-publication history for this paper can be accessed here:

http://www.biomedcentral.com/1471-2407/10/272/prepub
